# Accuracy of controlled attenuation parameter compared with ultrasound for detecting hepatic steatosis in children with severe obesity

**DOI:** 10.1007/s00330-020-07245-2

**Published:** 2020-09-10

**Authors:** Jurgen H. Runge, Jet van Giessen, Laura G. Draijer, Eline E. Deurloo, Anne M. J. B. Smets, Marc A. Benninga, Bart G. P. Koot, Jaap Stoker

**Affiliations:** 1 Department of Radiology and Nuclear Medicine, Amsterdam University Medical Centers, Location Academic Medical Center, University of Amsterdam, Amsterdam, The Netherlands; 2 Department of Pediatric Gastroenterology and Nutrition, Amsterdam University Medical Centers, Location Academic Medical Center/Emma Children’s Hospital, University of Amsterdam, Amsterdam, The Netherlands; 3 Amsterdam Reproduction & Development Research Institute, Amsterdam University Medical Centers, Location Academic Medical Center/Emma Children’s Hospital, Amsterdam, The Netherlands; 4 Amsterdam UMC, University of Amsterdam, Gastroenterology and Hepatology, Amsterdam Gastroenterology Endocrinology Metabolism Research Institute, Amsterdam, The Netherlands

**Keywords:** Elasticity imaging techniques, Non-alcoholic fatty liver disease, Child, Obesity

## Abstract

**Objectives:**

To determine the diagnostic accuracy of controlled attenuation parameter (CAP) on FibroScan^®^ in detecting and grading steatosis in a screening setting and perform a head-to-head comparison with conventional B-mode ultrasound.

**Methods:**

Sixty children with severe obesity (median BMI z-score 3.37; median age 13.7 years) were evaluated. All underwent CAP and US using a standardized scoring system. Magnetic resonance spectroscopy proton density fat fraction (MRS-PDFF) was used as a reference standard.

**Results:**

Steatosis was present in 36/60 (60%) children. The areas under the ROC (AUROC) of CAP for the detection of grade ≥ S1, ≥ S2, and ≥ S3 steatosis were 0.80 (95% CI: 0.67–0.89), 0.77 (95% CI: 0.65–0.87), and 0.79 (95% CI: 0.66–0.88), respectively. The AUROC of US for the detection of grade ≥ S1 steatosis was 0.68 (95% CI: 0.55–0.80) and not significantly different from that of CAP (*p* = 0.09). For detecting ≥ S1 steatosis, using the optimal cutoffs, CAP (277 dB/m) and US (US steatosis score ≥ 2) had a sensitivity of 75% and 61% and a specificity of 75% and 71%, respectively. When using echogenicity of liver parenchyma as only the scoring item, US had a sensitivity of 70% and specificity of 46% to detect ≥ S1 steatosis. The difference in specificity of CAP and US when using only echogenicity of liver parenchyma of 29% was significant (*p* = 0.04).

**Conclusion:**

The overall performance of CAP is not significantly better than that of US in detecting steatosis in children with obesity, provided that the standardized scoring of US features is applied. When US is based on liver echogenicity only, CAP outperforms US in screening for any steatosis (≥ S1).

**Key Points:**

*• The areas under the ROC curves of CAP and ultrasound (US) for detecting grade ≥ S1 steatosis were 0.80 and 0.68, respectively, and were not significantly different (p = 0.09).*

*• For detecting grade ≥ S1 steatosis in severely obese children, CAP had a sensitivity of 75% and a specificity of 75% at its optimal cutoff value of 277 dB/m.*

*• For detecting grade ≥ S1 steatosis in clinical practice, both CAP and US can be used, provided that the standardized scoring of US images is used.*

## Introduction

Due to the increasing prevalence of obesity, non-alcoholic fatty liver disease (NAFLD) has become the most common chronic liver disease in adults and children worldwide [1, 2]. The NAFLD spectrum ranges from simple steatosis to non-alcoholic steatohepatitis (NASH), fibrosis, and cirrhosis. The pooled prevalence of NAFLD in children ranges from 7.6% in general population studies to 34.2% in studies based on child obesity clinics [1, 3, 4]. Although simple steatosis is the most common and benign stage in children, advanced fibrosis is reported in 17% of children with elevated alanine aminotransferase (ALT) referred from primary care to liver centers after screening [5]. In addition, NAFLD increases the risk of developing type 2 diabetes and cardiovascular diseases at adult age [6, 7]. Timely detecting and staging of NAFLD is therefore of great importance, particularly in children who might be at a higher risk of complications in their lifetime given their longer life span [8–10].

Screening for NAFLD in children with obesity is propagated in most national and international obesity and hepatology guidelines [11]. However, the advised screening tools in these guidelines, serum ALT and conventional B-mode ultrasound (US), have a poor accuracy for detecting and grading steatosis [5, 12–20]. Magnetic resonance techniques, including magnetic resonance spectroscopy proton density fat fraction (MRS-PDFF), have excellent accuracy and reproducibility for detecting and grading liver steatosis in adults and children [21–26]. MRS-PDFF has been accepted as a non-invasive reference standard for quantifying hepatic steatosis in studies [23, 27, 28]. These MR techniques are not suitable for screening in daily clinical practice due to relatively high costs and dependence on patient compliance, which can be a concern in the pediatric population. Therefore, an easy-to-use, inexpensive, and accurate screening tool for NAFLD is still very much needed.

In recent years, the controlled attenuation parameter (CAP) became available on the FibroScan^®^ device (Echosens SA) for the detection of hepatic steatosis. CAP reflects the attenuation (loss of signal amplitude) of an ultrasound beam as it traverses tissue and is influenced by the amount of hepatic fat [29]. CAP uses standardized (controlled) settings, thereby minimizing user influence on the attenuation value. CAP is expressed as a number, whereas in conventional B-mode ultrasound, the grayscale values (which in part reflect the level of attenuation) are interpreted by an observer. These interpretations are subject to inter-observer variability. A second advantage of CAP is that FibroScan^®^ simultaneously measures liver stiffness to assess fibrosis, which is of great benefit when evaluating children with NAFLD.

The aim of this study was to determine the diagnostic accuracy of CAP in (a) detecting and (b) grading hepatic steatosis in children with obesity in a screening setting using MRS-PDFF as the reference standard. As a secondary aim, we performed a head-to-head comparison of the accuracy of CAP and US in detecting steatosis in children with obesity.

## Methods

### Study design and patients

This prospective study was registered in the Dutch Trial Register (NL4155). Participants were recruited from the outpatient obesity clinics of two hospitals in The Netherlands (Slotervaart Ziekenhuis and Amsterdam UMC), during a 4-year study period between 2014 and 2018. In The Netherlands, all children with obesity grade 3 (“severe obesity”) or grade 1 or 2 obesity with elevated glucose levels are referred to obesity clinics for evaluation of metabolic complications. Inclusion criteria were age 8–18 years and obesity (BMI z-score > 2). The exclusion criteria were presence of a liver disease other than NAFLD (viral/autoimmune hepatitis, Wilson disease, hemochromatosis, alpha-1 antitrypsin deficiency), known focal liver lesion(s) in the right liver lobe (proven with histology results or imaging), use of steatogenic drugs, and contraindications for MRI. Patients were consecutively included. All participants underwent conventional B-mode US of the liver, CAP measurement on FibroScan^®^, and MRS-PDFF at our tertiary hospital after a fasting period of at least 6 hours. The measurements were obtained during one or two visits, depending on the availability of MRI, FibroScan^®^, and US. The study was approved by the Medical Ethics Committee of the Amsterdam UMC, location Academic Medical Center. Written informed consent was obtained from the participants aged 12 years or older and/or their legal guardians. We followed the Standards for Reporting of Diagnostic Accuracy (STARD) guidelines in this study on the accuracy of CAP and US in detecting hepatic steatosis [30].

### Sample size calculation

Sample size calculations were performed for both primary and secondary outcomes; as the secondary outcome required a larger sample size, this is given here. Bohte et al previously showed that US had a sensitivity of 85% (95% CI: 77–91) and a specificity of 55% (95% CI: 46–65) for detecting any degree of steatosis in children with obesity [12]. Based on studies performed in adults, we hypothesized that CAP would have a specificity at least 15% higher than US. Using a McNemar test, we calculated that 26 non-steatotic subjects were required to reach a significant difference in specificity between US and CAP. Given the expected prevalence of steatosis (diagnosed on MRS-PDFF) of 50% [12], a sample size of 52 subjects was anticipated required. After 40 subjects were included, interim analysis revealed a lower prevalence of non-steatotic subjects than anticipated (43%). The number of required subjects was thereupon increased to 60 to allow more non-steatotic subjects to be included. Regardless of the final prevalence, study inclusion was stopped after 60 subjects had completed all study procedures.

### Clinical assessment

Physical examination included measurement of weight, height, body mass index (BMI), waist circumference, and blood pressure. The BMI z-score was calculated. The BMI z-score is the number of standard deviations (SD) from the mean on a standard BMI curve for age and gender. Children with a BMI z-score of > 2 (equals > 95th percentile) were considered to have obesity, and those with a BMI z-score of > 2.6 (equals > 99th percentile) were considered to have severe obesity [31, 32].

### Laboratory tests

Alanine aminotransferase (ALT) levels were obtained from the patients’ clinical record. Values obtained within 3 months to the study visit were used for analysis. No blood was sampled for the purpose of this study.

### Reference test: proton density fat fraction with magnetic resonance spectroscopy

All subjects underwent an MRI scan after fasting, consisting of localizers and several anatomic imaging sequences followed by the MRS-PDFF sequence. Briefly, a single breath-hold, multi-echo stimulated echoes acquisition mode (STEAM) ^1^H-MRS sequence was performed using a 20 × 20 × 20 mm^3^ voxel positioned in segment VI or VII of the liver. Further details on acquisition and post-processing have been described in a previous study in our hospital. MRI technicians were blinded to clinical information and CAP and US results. Steatosis grades 1 (S1), S2, and S3 were defined as an MRS-PDFF fat fraction of > 4.14%, > 15.72%, and > 20.88%, respectively. These thresholds were validated to correspond with steatosis grades 1, 2, and 3 of the NAFLD Activity Score (NAS) based on liver histology in adults in a previous study in our hospital [33].

### Index test 1: controlled attenuation parameter

The CAP measurement was obtained using the FibroScan^®^ 502 Touch Device with the 3.5-MHz M-probe. CAP was measured intercostally, in a midaxillary location, by one of two observers (J.R., a radiology resident who has performed more than 100 examinations, and B.K., a pediatric gastroenterologist who has performed more than 400 examinations). Both observers were blinded to US and MRS-PDFF results. Only CAP results with at least 10 valid measurements were analyzed.

### Index test 2: conventional B-mode ultrasound

The US assessment was performed using the ATL HDI 5000 IU22 and Epiq 5G (Philips Healthcare) equipped with 5-2 and 9-2 curved-array transducers. US was performed by one of two pediatric radiologists with 10 and 30 years of experience in pediatric US. Both were blinded to any other result. The “abdominal general” setting was used and gain and focus were manually adjusted, depending on patient habitus and beam attenuation. The following standardized views of the liver were obtained: transverse and longitudinal views of the right hepatic lobe, including the right kidney and diaphragm; a sagittal view of the left liver lobe; a view including the portal vasculature; and a view of the gallbladder region. To assess steatosis, the following four widely accepted items were evaluated by the radiologists: echogenicity of liver parenchyma, visualization of diaphragm, visualization of intrahepatic vessels, and visualization of posterior part of the right hepatic lobe [34]. Ultrasound images of these features were previously shown by Bohte et al [12]. A final qualitative score from 0 to 3 was given by the radiologists with respect to liver steatosis: the “ultrasound steatosis score” (US steatosis score; Table 1) [12]. We calculated the optimal US steatosis score to separate grade S0 steatosis from S1–S3, S0–S1 from S2–S3, and S0–S2 using the Youden index that maximizes sensitivity and specificity. We applied these optimal US steatosis scores to determine the accuracy of US in detecting ≥ S1, ≥ S2, and ≥ S3. In clinical practice, steatosis is often assessed based only on increased liver echogenicity, without taking other features into account. Therefore, we additionally determined the accuracy of US when using echogenicity of liver parenchyma as only scoring item and compared this with CAP.

**Table 1:** Scoring of hepatic steatosis with ultrasound (US steatosis score)

Score 0	Normal echogenicity of liver parenchyma
	Normal visualization of diaphragm and intrahepatic blood vessels
Score 1	Slightly increased echogenicity of liver parenchyma
	Normal visualization of diaphragm and intrahepatic blood vessels
Score 2	Markedly increased echogenicity of liver parenchyma
	Slightly decreased visualization of diaphragm and intrahepatic blood vessels
Score 3	Severely increased echogenicity of liver parenchyma
	No or severely decreased visualization of diaphragm and intrahepatic blood vessels and posterior part of the right liver lobe

### Statistical analysis

Descriptive statistics were used to analyze patients’ demographic, laboratory, and imaging data. We calculated the optimal cutoff value for CAP to separate grade S0 steatosis from S1–S3, S0–S1 from S2–S3, and S0–S2 from S3 using the Youden index. The diagnostic accuracy of CAP and US for grading steatosis was calculated, including sensitivity, specificity, positive predictive value (PPV), negative predictive value (NPV), positive and negative likelihood ratios (LR+, LR−), and receiver operating characteristic (ROC) curve with 95% confidence intervals. We compared the areas under the ROC curves (AUROC) of CAP and US using a (pairwise) comparison according to the method used by Hanley and McNeil [35]. We compared sensitivity and specificity of CAP and US in detecting ≥ S1 steatosis using the McNemar chi-square test. A value of *p* < 0.05 was considered a statistically significant difference. Statistical analyses were performed by using software (SPSS, version 22 [IBM]; GraphPad Prism 8.0 [GraphPad Software]; MedCalc Statistical Software, version 16.2.0 [MedCalc Software; https://www.medcalc.org; 2016]).

## Results

Sixty-two participants (34 males, 28 females) were consecutively included between October 2014 and December 2018. One participant refused MRI measurement and in one patient CAP measurement failed, most likely due to a large waist circumference. Patient characteristics are summarized in Table 2. The median age was 13.7 years (IQR 12.1–16.1). Median BMI z-score was 3.37 (IQR 3.01–3.98). Most participants were of Turkish or Dutch descent (38% and 27%, respectively). The median interval between MRS-PDFF and the index tests was 0 days (IQR 0–4)*.* Hepatic steatosis, defined as an MRS-PDFF fat fraction of > 4.14%, was present in 36/60 children (60%). Grade 2 steatosis was found in 7 patients and 8 patients had grade 3 steatosis based on MRS-PDFF.

**Table 2:** Patient characteristics

Demographic	
Age (years)	13.7 (12.1–16.1)
Female, *n* (%)	26 (43)
Ethnicity, *n* (%)	
Turkish	23 (38)
Caucasian	16 (27)
African	4 (7)
Surinamese	3 (5)
Asian	3 (5)
North-African	2 (3)
Other/unknown	9 (15)
Clinical	
Steatosis, *n* (%)	36 (60)
BMI z-score	3.37 (3.01–3.98)
Waist circumference, cm (IQR)	100 (90–121)
Biological data	
ALT, IU/L (IQR)	28 (19–39)
MRS-PDFF	
Fat fraction, % (IQR)	5.90 (2.12–15.38)
S0, *n* (%)	24 (40)
S1, *n* (%)	21 (35)
S2, *n* (%)	7 (12)
S3, *n* (%)	8 (13)
Ultrasound	
US steatosis score 0, *n* (%)	16 (27)
US steatosis score 1, *n* (%)	15 (25)
US steatosis score 2, *n* (%)	16 (27)
US steatosis score 3, *n* (%)	13 (21)

Continuous variables are expressed in median with interquartile range (IQR) in parentheses or *n* (%). ALT was measured in 40 patients. *ALT* alanine aminotransferase, *BMI* body mass index, *MRS-PDFF* magnetic resonance spectroscopy proton density fat fraction

### CAP versus ultrasound

**Fig. 1:**
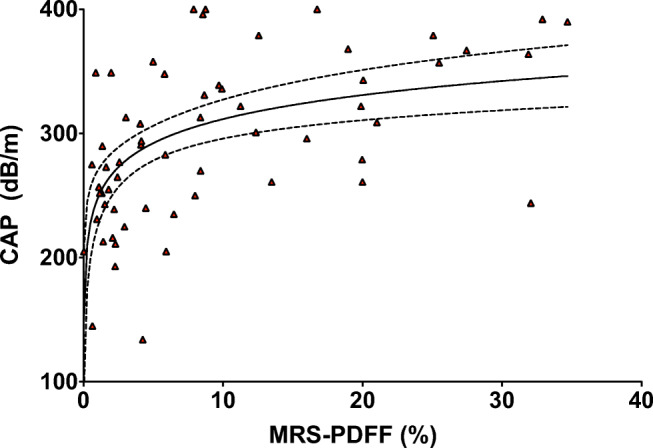
Scatterplot of CAP by MRS-PDFF. Scatterplot shows logarithmic relationship between CAP (dB/m) and MRS-PDFF (%). Solid and dashed lines represent lines of best fit with corresponding 95% confidence interval bands. *CAP* controlled attenuation parameter, *MRS-PDFF* magnetic resonance spectroscopy proton density fat fraction

**Fig. 2:**
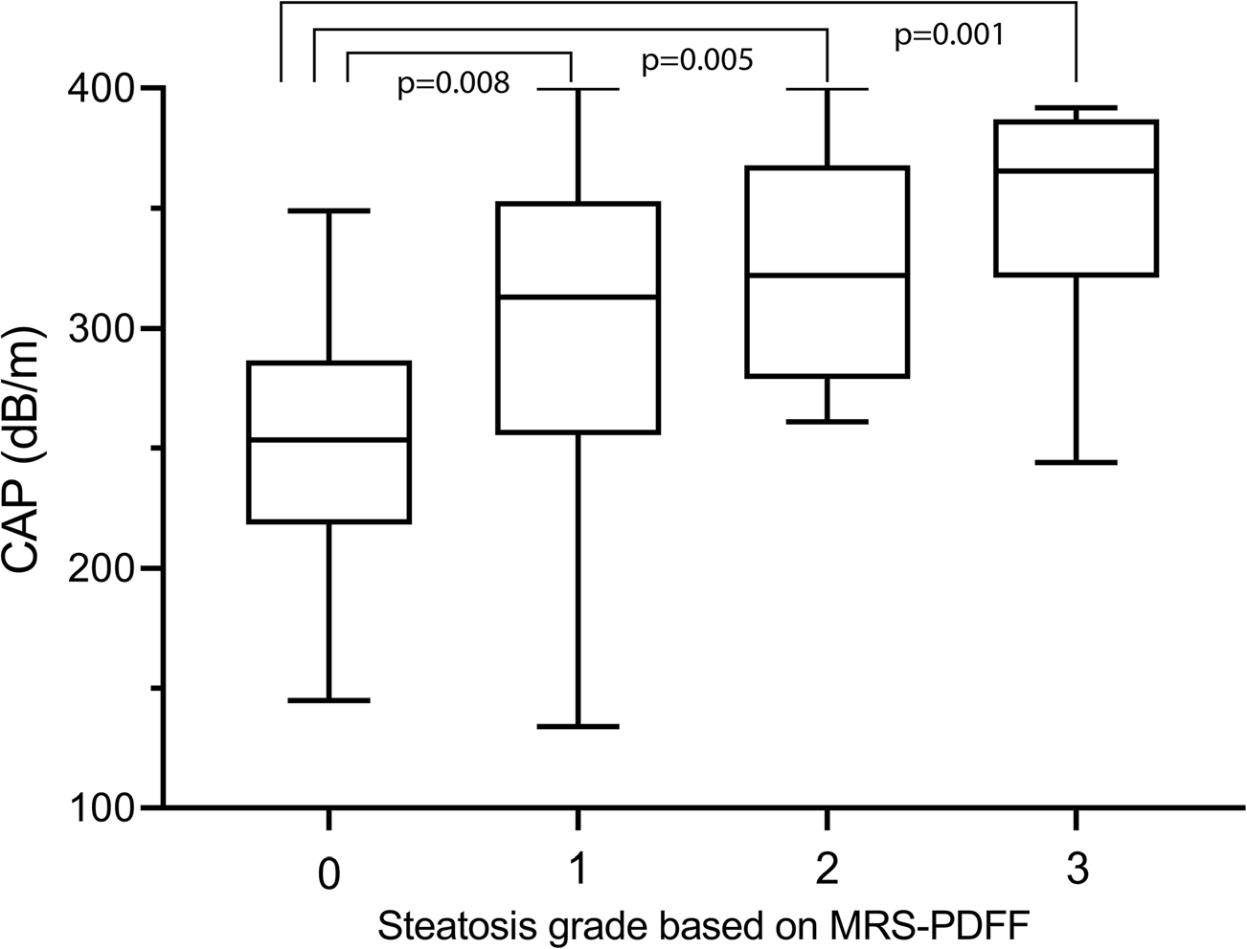
Box-and-whisker plot. Box-and-whisker plot of CAP according to steatosis grade at MRS-PDFF. Boxes show median and 25th and 75th percentiles, and whiskers show minimum and maximum values. Horizontal brackets indicate significant differences between steatosis grades. CAP controlled attenuation parameter, MRS-PDFF magnetic resonance spectroscopy proton density fat fraction

CAP and US were successfully obtained in 60 patients. Median CAP value was 253 dB/m (IQR 218–287) in children without steatosis versus 327 dB/m (IQR 272–368) in children with steatosis. The scatterplot in Fig. 1 shows the logarithmic relationship between CAP and MRS-PDFF. Figure 2 shows the distribution of CAP for the steatosis grades defined by MRS-PDFF. CAP differed significantly between grade S0 (no steatosis) and all other steatosis grades (S1 *p* = 0.008; S2 *p* = 0.005; S3 *p* = 0.001). CAP did not significantly differ between S1 and S2 (*p* = 0.54), S1 and S3 (*p* = 0.10), and S2 and S3 (*p* = 0.36).

**Fig. 3:**
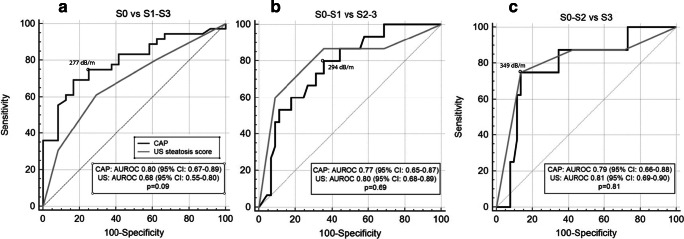
Comparison of ROC curves. Comparison of ROC curves for three cutoffs. **a** Grade S0 steatosis versus grades S1–S3. **b** Grades S0–S1 versus S2–S3. **c** Grades S0–S2 versus S3. Black and gray lines indicate CAP and US, respectively. Optimal cutoffs are calculated using the Youden index. AUROC area under the ROC curve, CAP controlled attenuation parameter, ROC receiver operating curve, US ultrasound

The AUROCs of CAP and US for the detection of grade ≥ S1, ≥ S2, and ≥ S3 steatosis were 0.80 versus 0.68, 0.77 versus 0.80, and 0.79 versus 0.81, respectively (Fig. 3). There was no significant difference in the AUROC of CAP and US for the detection of any stage of steatosis (S0 vs ≥ S1, *p* = 0.09) or the higher grades of steatosis.

Based on the Youden index, the optimal cutoff value for CAP for detecting ≥ S1 steatosis was 277 dB/m, yielding a sensitivity of 75% and a specificity of 75% (Table 3). At its optimal cutoff value (US score ≥ 2), US had a sensitivity of 61% and a specificity of 71% for detecting ≥ S1 steatosis (Table 3). The difference in sensitivity between CAP and US of 14% did not reach statistical significance (*p* = 0.23). When using echogenicity of liver parenchyma as only the scoring item, US had a sensitivity of 70% and specificity of 46% to detect ≥ S1 steatosis. The difference in specificity of CAP and US when using only echogenicity of liver parenchyma of 29% was significant (*p* = 0.04).

**Table 3:** Diagnostic accuracy of CAP and US

	AUROC	Cutoff	Sensitivity % (95% CI)	Specificity % (95% CI)	PPV % (95% CI)	NPV % (95% CI)	LR+ (95% CI)	LR− (95% CI)
CAP								
S0 versus S1–3	0.795 (95% CI: 0.671–0.888)	> 277 dB/m	75.0 (57.8–87.9)	75.0 (53.3–90.2)	81.8 (68.7–90.2)	66.7 (52.1–78.7)	3.00 (1.46–6.15)	0.33 (0.18–0.61)
S0–1 versus S2–3	0.774 (95% CI: 0.648–0.872)	> 294 dB/m	80.0 (51.9–95.7)	64.4 (48.8–78.1)	42.9 (32.0–54.5)	90.6 (77.4–96.5)	2.25 (1.41–3.59)	0.31 (0.11–0.87)
S0–2 versus S3	0.787 (95% CI: 0.662–0.882)	> 349 dB/m	75.0 (34.9–96.8)	84.6 (71.9–93.1)	42.9 (26.1–61.4)	95.7 (86.8–98.7)	4.88 (2.30–10.35)	0.30 (0.09–0.99)
US								
S0 versus S1–3	0.683 (95% CI: 0.550–0.797)	US score ≥ 2	61.1 (43.5–76.9)	70.8 (48.9–87.4)	75.9 (61.5–86.1)	54.8 (42.8–66.3)	2.10 (1.07–4.12)	0.55 (0.34–0.89)
S0–1 versus S2–3	0.801 (95% CI: 0.678–0.893)	US score ≥ 2	86.7 (59.5–98.3)	64.4 (48.8–78.1)	44.8 (34.3–55.8)	93.6 (79.7–98.2)	2.44 (1.57–3.79)	0.21 (0.06–0.77)
S0–2 versus S3	0.808 (95% CI: 0.685–0.898)	US score ≥ 3	75 (34.9–96.8)	86.5 (74.2–94.4)	46.2 (27.9–65.5)	95.7 (87.1–98.7)	5.57 (2.51–12.36)	0.29 (0.09–0.96)

Diagnostic accuracy of CAP and US to detect grade ≥ S1, ≥ S2, and ≥ S3 steatosis at their optimal cutoff values, determined using the Youden index. *AUROC* area under the ROC curve, *CAP* controlled attenuation parameter, *LR+* positive likelihood ratio, *LR−* negative likelihood ratio, *NPV* negative predictive value, *PPV* positive predictive value, *US* ultrasound

The optimal diagnostic accuracies of CAP and US to detect different stages (≥ S1, ≥ S2, ≥ S3) of steatosis using their optimal cutoffs are shown in Table 3.

## Discussion

This study reports on the accuracy of CAP and conventional B-mode US for detecting steatosis in children with obesity using MRS-PDFF as the reference standard. This is the first head-to-head comparison of CAP and US in children with obesity. The overall performance of CAP was not significantly better than that of US.

When screening for NAFLD, detecting steatosis is usually the first step to identify those patients that require additional testing to stage NAFLD, i.e., detecting and grading inflammation and/or fibrosis. The most widely used screening tools, serum ALT measurement and ultrasound, are limited by their mediocre accuracy which has led to the development of new techniques such as MRI-based measurements and CAP, an ultrasound-based measurement available on the FibroScan^®^ device. To evaluate the clinical value of new screening tools for NAFLD, it is most relevant to determine the accuracy of detecting any steatosis (≥ S1), since the degree of steatosis is not associated with the histological severity of liver disease and metabolic changes [36, 37]. For example, insulin resistance starts early on after hepatic triglyceride accumulation and is fully established at 1.5% (measured by ^1^H-MRS), which is well below the current cutoff point for the diagnosis of NAFLD [37].

Comparing the accuracy data from this study to those from previous studies is difficult as study populations and reference standards differ among studies. The performance of CAP for detecting ≥ S1 steatosis, reflected in the area under the ROC curve (0.80; 95% CI: 0.67–0.89), is comparable to that of adult studies: 0.85 in a meta-analysis in 2014 [38] and 0.77–0.96 in recent studies [33, 39–44]. In comparison to pediatric studies, Ferraioli et al found an AUROC of 0.84 (95% CI: 0.78–0.89) to detect ≥ S1 steatosis in a mixed population of children with and without obesity [45]. However, US was used as the reference standard which is less accurate than MRS-PDFF. Desai et al reported a higher performance of CAP: AUROC of 0.93 (95% CI: 0.87–0.99) and a sensitivity of 87% and specificity of 83% in detecting ≥ S1 steatosis at the optimal cutoff point, using biopsy as the reference standard [46]. This difference may be accounted to the difference in study population, as they included children with various liver diseases; only 14 out of 69 had NAFLD, and mean BMI z-score was 0.67 compared with a median BMI z-score of 3.51 in our study. Shin et al evaluated 86 children with and without obesity and also reported a higher accuracy of CAP: AUROC 0.94 (95% CI: 0.87–0.98) and a sensitivity of 99% and specificity of 80% in detecting ≥ S1 steatosis using MRI-PDFF as the reference standard [47]. Again the difference in BMI might explain the difference in performance, since only 17/83 children had severe obesity versus all children in our study. In agreement with our results, they found that discrimination between S1, S2, and S3 steatosis was suboptimal.

Thus far, different optimal CAP thresholds have been found in studies. In our study, a threshold of 277 dB/m was found to be most optimal to detect ≥ S1 steatosis which is comparable to the proposed thresholds from adult studies that range between 236 dB/m and 302 dB/m [33, 39, 40, 42, 43, 48]. This threshold is higher compared with the pediatric studies by Desai et al [46] and Shin et al [47]: 225 dB/m and 241 dB/m, respectively. The latter could be explained by the different study populations and underlying disorders, since in adults CAP values have been found to be significantly associated with BMI, waist circumference, and the studied liver disease, including higher values in NAFLD [41]. This implies that disease- or patient-group-specific thresholds are required. The threshold determined in this study needs validation in another population-based cohort of children with obesity.

To ensure a fair comparison between the accuracy of CAP (continuous variable) and US (categorical variable) in detecting ≥ S1 steatosis, we used the optimal thresholds for the detection of ≥ S1 steatosis of both tools. The US threshold in this study (US score ≥ 2) is in line with the previous reported pooled accuracy results of US [11]. In our study, CAP had a 14% higher sensitivity compared with US. This difference was not significant as our study was powered on detecting a difference of 15% in specificity in a cohort with lower anticipated prevalence of steatosis. To determine whether there is a small difference in accuracy between CAP and US, a larger study is needed. For clinical practice, this study implies that both CAP and US can be used for screening purposes depending on local availability and expertise.

When using ultrasound in clinical practice, steatosis is often considered present when only liver echogenicity is increased, and other US features (e.g., decreased visibility of intrahepatic vessels, decreased visualization of the diaphragm) are less frequently used or specifically mentioned in radiological reports. We found that when taking only liver echogenicity into account, specificity of CAP was 29% higher (*p* = 0.04). These findings underscore the relevance of evaluating more than just the increased echogenicity of the liver parenchyma when using US in children suspected to have steatosis.

The strength of this study is the use of MRS-PDFF as the reference standard as it has high accuracy in detecting and grading steatosis. In addition, the MRS-PDFF setting used in this study has been validated compared with histology [33]. Validation was performed in adults since validation with histology in a pediatric cohort of NAFLD is unfeasible due to ethical objections. Another strength is the inclusion of a multi-ethnic study population which is representative of the population targeted for NAFLD screening. This study therefore evaluates the usefulness of CAP and US in a setting identical to the real-life screening situation. A limitation of the current study is its sample size and the low number of children with higher steatosis grades, hampering the comparison of accuracy of CAP and US in grading steatosis. Also, we were not able to correlate CAP with metabolic factors, as no blood was drawn for the purpose of this study.

In conclusion, this study shows that CAP has a sensitivity and a specificity of both 75% at the optimal threshold of 277 dB/m. However, the overall performance of CAP compared with US for detecting steatosis in children with obesity is not significantly better, provided that the standardized scoring of US images is used. When US is based on liver echogenicity only, CAP had a 29% higher specificity and thereby outperforms US in detecting steatosis.

## Abbreviations

ALT: Alanine aminotransferase
AUROC: Area under the receiver operating characteristic (ROC) curve
BMI: Body mass index
CAP: Controlled attenuation parameter
LR-: Negative likelihood ratio
LR+: Positive likelihood ratio
MRS-PDFF: Magnetic resonance spectroscopy proton density fat fraction
NPV: Negative predictive value
PPV: Positive predictive value
US score: Ultrasound steatosis score
US: Ultrasound
